# Scrotal Abscess Precipitating Late Infection of a Malleable Penile Prosthesis: The Risk Never Evanesces

**DOI:** 10.1155/2016/3280418

**Published:** 2016-02-04

**Authors:** Osama Mustafa, Sultan Althakafi, Said Kattan, Mohamed Kattan, Naif AlHathal

**Affiliations:** ^1^College of Medicine, Alfaisal University, Riyadh, Saudi Arabia; ^2^Department of Urology, King Saud Medical City, Riyadh, Saudi Arabia; ^3^Department of Urology, King Faisal Specialist Hospital and Research Center, P.O. Box 3354, Riyadh 11211, Saudi Arabia

## Abstract

Although infrequent, infections represent the dreadful complication of penile prosthesis implantation. The incidence substantially decreases after a few infection-free postoperative months. We report herein a case of a very late penile prosthesis infection from a fistualizing scrotal abscess in a 67-year-old man. The patient presented with a one-month history of persistent penile-base discharge from a right hemiscrotal swelling. On examination, mild penile tenderness and a discharging penoscrotal-junction sinus were noted. Microbiological wound culture was positive for* Staphylococcus epidermidis*. Magnetic resonance imaging revealed this multiloculated fluid collection's communication with the right corporal body. Removal of the prosthesis was performed. Pathological evaluation of the dissected fistula was suggestive of acute on top of chronic inflammatory reactions. To our knowledge, this is the first report of a scrotal abscess leading to penile prosthesis infection 15 years after an uneventful implantation.

## 1. Introduction

The advances made in materials and designs of penile prosthetic implants have fortunately resulted in a dramatic reduction in the rate of mechanical failure and other complications [1, 2]. Infections, the grave complication of penile prosthesis implantation, have been relatively uncommon to a degree that may explain the current lack of management guidelines and practice recommendations by the pertinent professional bodies [3].

While erosions represent a well-recognized delayed complication of penile prosthesis implantation [4, 5], late-onset infections are particularly rare. Should any occur, the highest “vulnerability window” appears to span the first few postoperative months [6]. In fact, it was estimated that only 2.6% of all penile prosthetic infections may occur after 5 years of implantation [6]. We report a rare case of scrotal abscess leading to late-onset infection 15 years after an uncomplicated penile prosthesis implantation.

## 2. Case Presentation

A 67-year-old gentleman, who underwent malleable penile prosthesis placement 15 years ago for diabetes-induced refractory erectile dysfunction, presented with a one-month history of an on-and-off, thin, blood-mingled penile-base discharge. Three months earlier, the patient experienced a constant, aching pain, which was moderate in severity and associated with a well-defined right hemiscrotal swelling. He denied having hematuria or lower urinary tract symptoms. He reported no recent history of urologic procedures, indwelling catheter use, local trauma, or intracavernous injections. He was seen on multiple occasions at a local hospital, from which he was discharged on antipyretic analgesics and several courses of antibiotics.

The patient's past medical history was significant for long-standing diabetes mellitus, tooth extraction, and left maxillary antral biopsy for suspected nasopharyngeal carcinoma, which was diagnosed and treated with chemotherapy and radiotherapy two years prior to presentation. On physical examination, he was afebrile. Genitourinary examination revealed a nontender swelling, 30 mm in diameter, at the right penoscrotal junction with a discharging sinus tract. The discharge was turbid and slightly viscous. Mild penile shaft tenderness was noted. The rest of the examination was unremarkable.

**Figure 1:**
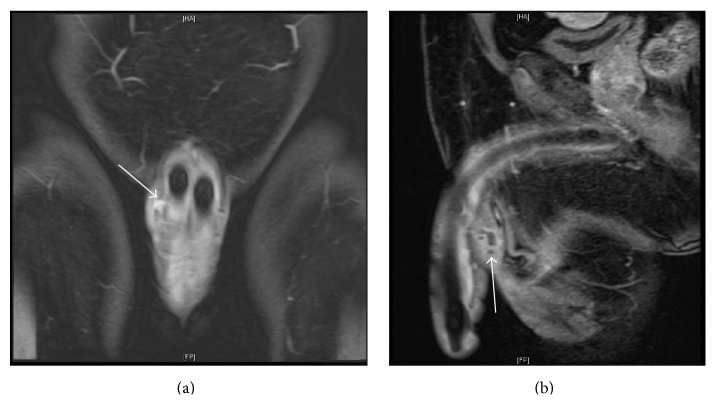
Coronal (a) and sagittal (b) MRI views of the collection revealing its multiloculated structure and communication with the right corporal body (arrows).

On blood workup, no relevant aberrations were identified. Urinalysis values were within normal limits, and urine culture showed no evidence of infection. Microbiological culture of the wound was positive for* Staphylococcus epidermidis*. Sonographic evaluation of the swelling demonstrated a ventral fluid collection with thick vascular periphery lying in close proximity to the right corporal body. Measuring around 37 × 14 mm^2^ in its largest dimensions, the multiloculated collection showed central fluid signal intensity and peripheral rim enhancement on a contrast-enhanced MRI scan, which revealed this collection's posterior communication with the right corpus cavernosal body (Figure 1). The urinary bladder, prostate, and testes were unremarkable.

The patient was counseled on the treatment options and the potential advantages and disadvantages of rescue procedures. Intraoperative flexible cystoscopic examination showed normal urethra and bladder mucosa. Meticulous dissection of the fistula tract revealed its connection with the right corporal implant. As the right corpus cavernosum was opened, a copious, opaque, coffee-ground exudate was discharged. Moreover, a communication between the left and right corpora was identified. Removal of both corporal prosthetic implants, along with copious irrigation with antibiotics, chlorhexidine, and polyvinylpyrrolidone solutions, was then performed. At the discretion of the operating surgeon, no salvage procedure was performed. Pathological evaluation of the excised fistula showed granulation tissue with acute and chronic inflammatory reactions consistent with fistula tract formation. The patient was discharged home on the fourth postoperative day, and his convalescence was uneventful.

## 3. Discussion

Although penile prosthesis infections represent an infrequent but well-recognized complication in early postoperative period, late-onset infections are exceedingly rare. To our knowledge, scrotal abscess resulting in a 15-year-postimplantation penile prosthesis infection is unique in the literature. The routes through which microorganisms gain access to the prosthesis differ between early and late infections. Early infections are likely to result from intraoperative seeding, while late infections may arise from hematogenous spread postoperatively [6]. Other possible means of access in late infections include intracavernous injections, trauma, and rarely fistulas [7]. Fistulization represents a distorted healing process of an otherwise natural inflammatory response to a local insult; locally active inflammation, neoplasm, infection, intraoperative damage, mechanical failure, and trauma may well stand behind fistula formation [7, 8]. In this case, given the history of hemiscrotal swelling preceding the penile infection, it is likely that the inflammatory process was instigated by the scrotal abscess, eroding into the skin in one direction and into the right corpus cavernosum in the other direction—forming a corporal-cutaneous tract.

Once in place, such tracts result in the drainage of the abscesses and, thus, facilitate clearing the infection. However, if the communication persists, it may function as a conduit that allows the migration of skin microbiota up to the prosthesis, leading to colonization and infection. Indeed, the microbiology evaluation in this case, which was indicative of* Staphylococcus epidermidis* infection, is suggestive of the local extension of this ubiquitous skin colonizer. While several factors may permit sustainability of such inflammatory tracts, repeated infections and microtrauma are likely culprits. Unresolving or undertreated infections (as that of a nondrained abscess) and minimal yet repetitive trauma resulting from the malleable prosthesis's friction with, and compression of, the surrounding tissue may have contributed to the enduring cascade of inflammatory reactions. The latter process was suggested to underlie, at least in part, the erosion of a malleable prosthesis into the bladder which occurred 5 years after implantation [4].

Alternatively, a subtle prosthetic infection resulting from the dental and maxillofacial procedures the patient underwent at an earlier point may be the underlying mechanism [9]. However, the nearly avascular, fibrous pseudocapsule that typically develops around the implanted cylinders makes the blood a less likely route of infection.

Notably, no dramatic clinical manifestations of the infection were observed in this patient. Prior treatment and the presence of long-standing diabetes mellitus may have resulted in the absence of an overt clinical reaction. In a temporally comparable poststernotomy mediastinal infection, Oh et al. suggested a tendency toward lacking frank inflammatory clinical features, such as fever, chills, and pain, in late-onset infection presentations [10].

Salvage procedures have gained popularity in recent years. A retrospective multi-institution review of 58 salvage procedures suggested a success rate of 93% among the infected inflatable penile prostheses that were replaced with malleable implants [11]. While this procedure was considered in this case, the patient did not express the interest to have it, leaving it to the discernment of the operating surgeon who did not deem it necessary.

## 4. Conclusion

Albeit rare, a remarkably late penile prosthetic infection may occur. Scrotal abscess may lead to the occurrence of such late infections. We presented a patient with a scrotal abscess leading to penile prosthesis infection as late as 15 years after successful implantation.
